# Digital plagiarism - The web giveth and the web shall taketh

**DOI:** 10.2196/jmir.2.1.e6

**Published:** 2000-03-31

**Authors:** John M Barrie, David E Presti

**Affiliations:** ^1^Biophysics GroupUniversity of California at BerkeleyBerkeleyCA 94720-3200USA; ^2^Department of Molecular and Cell BiologyUniversity of California at BerkeleyBerkeleyCA 94720-3200USA

**Keywords:** Ethics, Manuscript, Internet, World Wide Web

## Abstract

Publishing students' and researchers' papers on the World Wide Web (WWW) facilitates the sharing of information within and between academic communities. However, the ease of copying and transporting digital information leaves these authors' ideas open to plagiarism. Using tools such as the Plagiarism.org database, which compares submissions to reports and papers available on the Internet, could discover instances of plagiarism, revolutionize the peer review process, and raise the quality of published research everywhere.

After obtaining information regarding a 1996 finding that Polish authors had plagiarized the work of Danish researcher Jan Fallingborg, Marek Wronski (a cancer researcher from New York's Staten Island University Hospital) conducted a Medline search which revealed 30 additional manuscripts that contained passages allegedly taken from prior publications of other researchers [1]. This could have been just another exposure of a plagiarizing scientist [2,3] except that it was uncovered by the use of new, Internet-based technology. Fallingborg and Wronski both used a simple search function to sift through the massive amount of information contained within Medline (a database of biomedical references and abstracts) to locate acts of gross plagiarism that may never have been discovered otherwise. The ability to utilize powerful computers to mine large electronic databases for instances of plagiarism could revolutionize the peer review process and raise the quality of published research everywhere. As with most technologies, there are also some glaring negatives, in that digital information is easily copied and transported, and thus more available to plagiarize.

Medline is just one example of an Internet tool that facilitates previously unheard of levels of information dissemination. The large Internet search and navigational guides such as Yahoo!, Altavista, and Excite [4] convey Internet-based information to enormous audiences. For example, each month about 25 million users of the World Wide Web (WWW, one modality of the Internet) access information from Yahoo! at an average rate of 50 million web pages of information viewed per day, or 1.5 billion pages viewed per month [5]. This is a larger audience than magazines such as Newsweek, Time, and Life. These navigational guides also direct a subpopulation of the electronic community to numerous Internet locations that provide free access to very large databases containing thousands of academic term papers. Evil House of Cheat, Cheater.com, and School Sucks [6] are just a few locations which offer term papers to be used as "reference materials, research guides, or as educational resources."

The proliferation of sites specializing in the electronic commerce of academic papers is based on the simple truth that there is a sufficient demand for such work. Evil House of Cheat receives several thousand visits per day [7] and claims that over 11,000 students have benefited from their services [6]. Cheater.com claims to have over 30,000 members and adds approximately 100 new essays to its database each week [6]. School Sucks has received almost 1.6 million visits since its inception in the summer of 1996 [6]. Notwithstanding the warnings against plagiarism that greet their clientele, these web entities are supplying term papers to a student population that could choose to ignore such advice.

In a 1991 study of 15,904 students taken from 31 top U.S. universities, Rutgers University professor Donald McCabe found that 66% cheated at least once and that 12% were regular cheaters (four or more times) [8]. A study in Psychological Record found that 36% of the undergraduate participants had plagiarized written material and a significant number of the participants could not even determine what plagiarism was [9]. Nor are graduate and post-doctoral students immune to plagiarism, especially when it serves the purpose of obtaining funding or publishing a manuscript [10].

The problem concerning free Internet access to student term papers is not nearly so simple because it is not limited to dedicated Internet paper databases. Before the existence of Internet term paper providers, we confronted this very problem. In the 18 October 1996 issue of Science we reported on our model for utilizing the World Wide Web as an adjunct to education in a neurochemistry class (at that time 123 students; currently 320 students) at the University of California at Berkeley [11]. The final course project required that each student submit a 10-page manuscript through our Web site. Every manuscript was anonymously posted to the Internet, electronically peer-reviewed by two other students, and the reviews were then anonymously placed on the Web site alongside each paper. From our largely Internet-naive student population there was a 98.4% completion rate for this assignment, indicating that the use of Internet technology was not problematic. What was potentially problematic concerned the placement of high-quality term papers onto the WWW, where any person could view and copy a manuscript at will. Our temporary solution to the dilemma of free access versus plagiarism potential was to password-protect the manuscript domain of the course web site so that only students from that class could read their peers' papers. Password protection may seem antithetical to the idea of sharing information within and between academic communities, but the alternative is an Internet link from sites advertising free student term papers to the university web sites containing such papers and other academic essays [12].

Current solutions available for instructors include guidelines for spotting plagiarism, searching the Internet for similar papers, and attempting to instill a firm sense of ethics in their students. These solutions are incomplete when applied to the modern advancements in the technology of information dissemination. Our proposed solution has been to construct an archive of manuscripts (from previous classes and gathered from the Internet) that allows for a computer-based, digital check of originality for all newly submitted manuscripts. This experiment resulted in no manuscript being recycled from previous science courses, and yielded increased term paper quality levels. What it didn't address is the real possibility that a course manuscript could be used for a different class. We have now addressed this problem with the creation of Plagiarism.org. This Internet service allows any instructor or student to check our database for cases of gross term paper plagiarism by tracking and "finger-printing" those term papers already in the public domain and from other classes. Manuscript "finger-prints" are statistically compared and degrees of originality are computed.

We have successfully utilized the power of the WWW to allow students to share information and ideas at levels not previously achievable in the classroom. Now we have harnessed that power to insure that reference materials from Internet paper databases and from other university classes are used appropriately. The WWW has increased in size by 480% in the 18 months between October 1996 and April 1998 [13], and our three-year experiment checking term papers has necessarily gone from an experiment to a reality. This technology gives us a glimpse of the direction that education is evolving in the digital age.
